# Contribution of Neuronal and Glial Two-Pore-Domain Potassium Channels in Health and Neurological Disorders

**DOI:** 10.1155/2021/8643129

**Published:** 2021-08-12

**Authors:** Yuncheng Luo, Lu Huang, Ping Liao, Ruotian Jiang

**Affiliations:** Laboratory of Anesthesia and Critical Care Medicine, National-Local Joint Engineering Research Center of Translational Medicine of Anesthesiology, West China Hospital, Sichuan University, Chengdu 610000, China

## Abstract

Two-pore-domain potassium (K2P) channels are widespread in the nervous system and play a critical role in maintaining membrane potential in neurons and glia. They have been implicated in many stress-relevant neurological disorders, including pain, sleep disorder, epilepsy, ischemia, and depression. K2P channels give rise to leaky K^+^ currents, which stabilize cellular membrane potential and regulate cellular excitability. A range of natural and chemical effectors, including temperature, pressure, pH, phospholipids, and intracellular signaling molecules, substantially modulate the activity of K2P channels. In this review, we summarize the contribution of K2P channels to neuronal excitability and to potassium homeostasis in glia. We describe recently discovered functions of K2P channels in glia, such as astrocytic passive conductance and glutamate release, microglial surveillance, and myelin generation by oligodendrocytes. We also discuss the potential role of glial K2P channels in neurological disorders. In the end, we discuss current limitations in K2P channel researches and suggest directions for future studies.

## 1. Introduction

Ion channels in the cell membrane, particularly potassium channels, play a vital role in the resting membrane potential of neurons and in the transmission of action potentials [1–5]. An appropriate resting membrane potential requires a leaky K^+^ current, and two-pore-domain potassium (K2P) channels appear crucial in providing such current. These channels remain open (“leaky”) across the range of physiological voltages, as well as during action potentials [6–8].

The first mammalian K2P channel, TWIK-1, was identified in humans in 1996 [9], and since then, another 14 members sharing a similar structure have been identified and organized into six subfamilies (TWIK, TREK, TASK, TALK, THIK, and TRESK) [7, 8]. In contrast to other K^+^ channels, which feature one pore domain per subunit, K2P channels contain two pore domains per subunit. TWIKs give rise to a weakly inwardly rectifying K^+^ current, while TREKs are sensitive to mechanical and thermal stimuli as well as lipids. TASKs and TALKs respond strongly to changes in extra- or intracellular pH, while THIKs are sensitive to halothane.

K2P channels are widely expressed in the nervous system, including the dorsal root ganglion (DRG), trigeminal ganglion (TG), spinal cord, cerebrum, and cerebellum [10, 11]. In the peripheral nervous system, K2P channels are expressed in sensory neurons of the DRG and TG. In the brain, TWIK-1, TREK-1, TREK-2, TRAAK, TASK-1, and TASK-3 are preferentially expressed in specific regions. K2P channels are also expressed in various patterns in different cell types in other brain regions.

Perhaps the best-understood functions of K2P channels are passive K^+^ conductance and maintenance of resting membrane potential. Activation of K2P channels leads to the extrusion of K^+^ into the extracellular space, hyperpolarizing the neuronal membrane and dampening neuronal excitability. Various endogenous and exogenous stimuli can regulate the activity of these ion channels, including membrane stretching, voltage, temperature, extra- and intracellular pH, phospholipids, and other intracellular signaling molecules generated through the activation of G protein-coupled receptors (GPCRs) [12].

Some K2P members are expressed not only in neurons but also in glial cells. Glia are nonneuronal cells in the nervous system that do not produce electrical impulses. Instead, they maintain homeostasis of certain ions, particularly K^+^, in the extracellular environment. In the brain, TASK-1 has been detected in astrocytes and oligodendrocytes, while TWIK-1 and TREK-1 are expressed in astrocytes, and THIK-1 was identified in microglia [13].

This review summarizes recent studies of K2P channel functions in neurons and glia of the central and peripheral nervous systems. These studies have relied primarily on genetic and pharmacological tools to elucidate channel function. We focus on how K2P channels in neurons help maintain resting membrane potential and regulate excitability, and on how K2P channels in glia help maintain potassium homeostasis.

## 2. K2P Channels and the Excitability of Neurons

### 2.1. Peripheral Nervous System (PNS)

**Figure 1:**
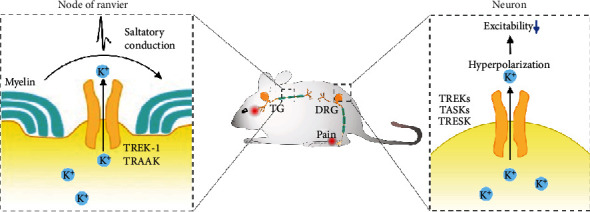
Functions of K2P channels in the peripheral nervous system. Left: TREK-1 and TRAAK are enriched in nodes of Ranvier on myelinated afferent nerves. The channels rapidly regenerate action potentials, allowing fast action potential conduction along the nerve. Right: K2P channels are expressed in neurons of the dorsal root ganglion and trigeminal ganglion. They extrude K^+^ and hyperpolarize the membrane of neurons at rest, decreasing their excitability.

Some K2P channels mediate nociception and pathological pain, and knocking them out in mice increases sensitivity to noxious stimuli [14, 15]. These findings indicate a strong association between K2P channels and neuronal excitability. Indeed, animal models of pathological pain show downregulation of K2P channels in the DRG or TG, as well as increased excitability of nociceptive neurons [16] (Figure 1).

#### 2.1.1. TWIK-Related Spinal Cord K^+^ Channels (TRESKs)

TRESK was first found in the human spinal cord, then discovered in mouse cerebrum [17]. In DRG neurons, this channel contributes strongly to background K^+^ currents: mutating Gly339 to Arg in TRESK in mice significantly reduced standing outward current in DRG neurons, without affecting resting membrane potential [18, 19]. At the same time, the mutation increased excitability of DRG neurons: it reduced the rheobase to elicit an action potential, and it increased the rate of action potential firing [18, 20, 21]. Knocking out TRESK in mice sensitized the animals to mechanical stress and cold, based on assessments of inflammatory and neuropathic pain responses, without substantially affecting their sensitivity to heat [20, 21]. Bone metastasis in mice has been linked to depolarization of nociceptive neurons and to pain hypersensitivity, which were partially reversed by overexpressing TRESK in DRG neurons and exacerbated by knocking down TRESK [22]. Similarly, treating isolated DRG neurons with the TRESK inhibitor isobutylalkenyl amide depolarized the cells and evoked action potential [23].

TRESK has been linked to migraine pathophysiology, and a frameshift mutation (F139WfsX24) has been identified in patients suffering from migraine with aura [24]. In mice, this frameshift mutation showed a dominant-negative effect of reducing the rheobase and increasing the rate of action potential firing in TG, without altering resting membrane potential [25]. The frameshift may cause these effects by giving rise to a truncated protein, TRESK-MT2 [26]. Expression of TRESK-MT2 in TG neurons of mice inhibited TREK-1 and TREK-2, reduced the rheobase, and increased the firing rate, indicating greater excitability. Using CRISPR-Cas9 to correct the frameshift in induced pluripotent stem cells from migraine patients reversed the hyperexcitability of the resulting differentiated nociceptors. Activating TRESK using cloxyquin in a mouse model of migraine alleviated pain hypersensitivity to mechanical and thermal stimuli [27]. Future studies are needed to examine coexpression of TRESK and TREK, to determine whether endogenous levels of TRESK-MT2 in human nociceptors inhibit TREK-1/2, and to evaluate the contribution of TREK-1/2 to migraine pathogenesis.

TRESK is also expressed in the nodose ganglia (NG), and NG neurons in diabetic rats show hyperpolarization as well as upregulated TRESK relative to NG neurons in healthy controls. [28, 29]. Silencing TRESK in diabetic animals partially restored normal excitability of NG neurons and thereby the animal's sensitivity to satiety signals [28, 29].

The fact that TRESK is expressed primarily in sensory neurons makes it a potential target for treating allodynia and migraine. Developing specific activators of this channel may expand on the range of analgesic agents currently available.

#### 2.1.2. TREKs (TWIK-Related K^+^ Channels)

TREKs belong to another subfamily of K2P channels comprising TREK-1, TREK-2, and TRAAK. TREKs are sensitive to membrane stretching, pH, arachidonic acid, and temperature, making them polymodal pain perception channels. In the DRG, TREKs are expressed in small-diameter neurons and to a small extent in medium- and large-diameter neurons [30]. Knocking out TREK-1, TREK-2, or TRAAK from mice rendered them more sensitive to thermal and mechanical stimuli [31–33], which indicated enhanced neuronal excitability. Downregulating TREK-2 in C-fiber nociceptors depolarized the membrane by about 10 mV and increased spontaneous firing frequency. Downregulating TREK-2 also increased spontaneous pain behavior in a mouse model of complete Freund's adjuvant (CFA) [34].

Activating TREKs using the neuroprotective agent riluzole hyperpolarized the resting membrane in the superior cervical ganglion [35, 36]. Similarly, activating TREK-1/2 in DRG neurons with BL-1249 or GI-530159 hyperpolarized the resting membrane and reduced firing frequency [37]. These results suggest that TREK activation in the peripheral nervous system can alleviate polymodal pathological pain, but the selectivity of those TREK activators has not been rigorously established. Potentially more reliable support for the therapeutic potential of TREK activation comes from studies with the recently developed TREK-1/2 activator C3001a [38]. C3001a was found to activate TREK-1 with a half-maximal effective concentration (EC_50_) of 12.81 *μ*M, and it activated TREK-2 with similar efficacy, while it did not appreciably activate TRAAK or other human K2P channels (TASK-3, TASK-1, TRESK, and THIK-1). Applying c3001a to dissociated DRG neurons produced weak hyperpolarization, increased the rheobase, and reduced the number of action potentials in small-sized neurons [38]. Applying the compound to mice with spared nerve injury or complete Freund's adjuvant alleviated spontaneous pain and cold hyperalgesia. The compound also alleviated mechanical allodynia and inflammation in a mouse model of pancreatitis. Thus, activating TREKs may be a useful therapeutic strategy.

TREK-1 and TRAAK are enriched at nodes of Ranvier on myelinated afferent nerves. These nodes display unusual action potentials and quite leaky K^+^ conductance that is thermo- and mechanosensitive. The nodes of Ranvier on nearly all trigeminal A*β*-afferent nerves express both TREK-1 and TRAAK [39]. Knocking down or pharmacologically inhibiting either channel widened action potentials at the nodes, increased nodal membrane input resistance, and depolarized the resting membrane. Thus, these channels are probably required for rapid action potential repolarization at the nodes of Ranvier. The channels also permit rapid, high-frequency action potentials along myelinated afferent nerves. Consistent with these ideas, knocking down TREK-1 or TRAAK in TG of rat significantly reduced tactile responses, suggesting sensory behavioral deficit [39]. These channels may play similar roles in other parts of the mammalian nervous system that contain rapidly conducting somatosensory afferent nerves or motor nerves. Further study is needed to determine whether other K2P channels are also involved in rapid action potential conduction at nodes of Ranvier in myelinated nerves in the peripheral and central nervous systems.

#### 2.1.3. TWIK-Related Acid-Sensitive K^+^ Channels (TASKs)

TASK-1 and TASK-3 belong to a subfamily of K2P channels sensitive to changes in extracellular pH [40], and they are expressed in DRG [11, 41]. TASK-3 is primarily expressed in small-sized DRG neurons, where it colocalizes with TRMP8, TRPV1, and tyrosine hydroxylase, suggesting selective expression in nociceptive neurons [42]. In the peripheral nervous system, TASK-3 is expressed in TRPM8-positive neurons, and knocking it out from mice rendered them hypersensitive to cold, and this effect was associated with depolarization of the resting membrane and increased amplitude of action potentials in TRPM8-positive neurons [42].

More details about the *in vivo* function of TASK-3 under physiological and pathological conditions began to emerge with the structure-based design of a selective TASK-3 activator, CHET3 [43]. Administering CHET3 to animal models of acute and chronic pain alleviated spontaneous pain as well as hyperalgesia in response to cold, heat, and mechanical stress. Applying CHET3 to isolated DRG neurons increased the rheobase and decreased action potential frequency, without altering resting membrane potential [43]. These genetic and pharmacological studies reveal a role for TASK-3 in membrane excitability of nociceptive neurons.

Since TASK-3 is expressed at much higher levels in the TG than DRG [43], future research should evaluate the potential role of TASK-3 in trigeminal diseases and facial sensation.

### 2.2. Central Nervous System

**Figure 2:**
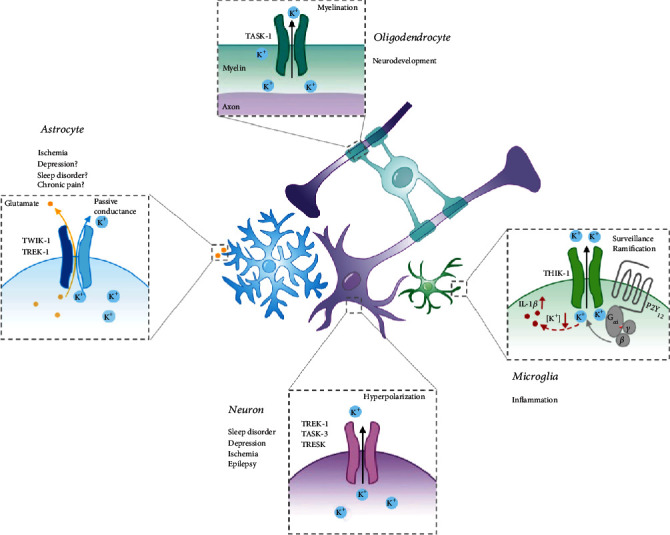
Roles of K2P channels in the central nervous system. K2P channels regulate neuronal excitability and contribute to many normal and disease processes, including sleep, epilepsy, ischemia, and depression. In glia, K2P channels maintain glial membrane potential and are involved in astrocytic passive conductance and glutamate release, microglial surveillance, myelin generation by oligodendrocytes, and K^+^ homeostasis.

In the central nervous system, mRNAs encoding K2P channels have been detected in the cerebrum, cerebellum, brainstem, and spinal cord [10, 11]. Neuronal K2P channels have been implicated in chronic pain [44], ischemia, epilepsy [45], sleep disorder, and major depressive disorder [46] (Figure 2).

#### 2.2.1. TRESK

High expression of TRESK has been found in several areas of the central nervous system, including the cortex, periaqueductal gray (PAG), and dorsal horn of the spinal cord [47]. Spinal nerve ligation in mice to induce neuropathic pain is associated with TRESK upregulation in the superficial dorsal horn [48], and overexpressing TRESK in such animals alleviated their hyperalgesia, inflammation, and neuronal apoptosis [49, 50]. These results suggest that TRESK in the spinal cord plays an essential role in pain perception, which needs to be confirmed in electrophysiological studies.

Recent evidence suggests that TRESK helps regulate nocturnal dynamics of the suprachiasmatic nucleus (SCN) and light-adaptive responses [51]. The SCN is the key circadian pacemaker, which synchronizes internal circadian rhythms to the external day-night cycle. TRESK expression increases in the early evening and remains high throughout the night, suggesting a rhythmic expression pattern. In the animal study, neurons at rest in the SCN of control animals were more depolarized during the day than at night, whereas the same neurons in animals lacking TRESK remained constantly in a depolarized state, and their nocturnal spike rate was higher than in controls. In addition, knocking out TRESK led to a much weaker Ca^2+^ response to glutamate [51].

#### 2.2.2. TREKs

TREK-1 and TRAAK are widely expressed across the central nervous system, with TREK-1 most abundant in the striatum, cortex and hippocampus. TRAAK is most abundant in the cortex. TREK-2 is restricted to the cerebellar granule cell layer [11, 52, 53].

*(1) TREK-1*. TREK-1 can regulate the excitability of neurons, but its precise roles may depend on the type of neuron. In pyramidal neurons of the CA1 region of the hippocampus, TREK-1 deficiency depolarized resting neurons, reduced the rheobase, and increased action potential frequency [54]. Similarly, in serotonergic neurons of the dorsal raphe nucleus (DRN), TREK-1 knockout increased discharge frequency in mice [55]. The channel does not, however, appear to affect resting membrane potential of GABAergic neurons in the striatum [56]. Treating brain slices with the endogenous TREK-1 inhibitor spadin or the inhibitor SID1900 increased the firing rate of serotonergic neurons in the DRN [57, 58]. Spadin also depolarized cortical neurons at rest [59]. The various effects of TREK-1 have been implicated in nervous system disorders as described below:

1. *Depression*: knocking out TREK-1 from several mouse models of depression alleviated depression symptoms, as measured in the forced swimming test, tail suspension test, conditioned suppression of motility test, and learned helplessness test [55]. In fact, the knockout animals showed similar behavior as wild-type mice treated with the antidepressants fluoxetine or paroxetine. Knockout was associated with higher firing rates of serotonergic neurons, which likely increased the release of serotonin into target structures. Mice lacking TREK-1 were insensitive to selective serotonin reuptake inhibitors, suggesting that the efficacy of such inhibitors involves TREK-1 inhibition [55]. Indeed, these inhibitors appear to inhibit TREK-1 in a dose-dependent manner [55, 60]. Both spadin and SID1900 enhanced the excitability of serotonergic neurons in mice, alleviating depressive symptoms [57, 58]. TREK-1 knockdown in hippocampal neurons alleviated depressive symptoms in a mouse model of lipopolysaccharide-induced depression [61]. Consistent with the studies on rodent models, four single nucleotide polymorphisms in TREK-1 were also identified in some patients with treatment resistance in major depressive disorders [62]

These observations suggest that TREK-1 may be a useful therapeutic target in depression. Future study is needed to determine whether TREK-1 actively regulates serotonin release and is involved in the mechanism of action of selective serotonin reuptake inhibitors.

(2) *Ischemia and seizure*: knocking out TREK-1 from mice increased their risk of ischemia due to transient bilateral occlusion of the common carotid arteries or due to occlusion of the aortic arch and left subclavian artery [56]. This ischemia was associated with higher mortality. Knockout also increased risk of epileptic seizures triggered by kainite or pentylenetetrazol; the mutant animals showed increased morbidity as well as increased spike amplitude and frequency, based on electroencephalography [56]. Administering spadin to mice did not reduce infarct size following focal ischemia or alleviate kainate-induced seizures [63]

TREK-1 in serotonergic neurons of the dorsal raphe has recently been implicated in the circadian photoperiod [64]. Longer photoperiods may reduce TREK-1 function, since pharmacological inhibition of TREK-1 significantly increased spike frequency in animals exposed to short or equinox photoperiods but not in animals exposed to long photoperiods, which expressed lower levels of the channel [64].

*(2) TREK-2/TRAAK*. TREK-2 is a downstream mediator of GABA_B_ receptors in neurons of the entorhinal cortex, giving the channel a strong influence over spatial learning. Treating the stellate neuron of the entorhinal cortex with the GABA_B_ receptor activator baclofen activated TREK-2, which blocked action potentials and hyperpolarized the membrane through mechanisms involving the G_ai_, G_ao_, and PKA pathways [65]. This decrease in neuronal excitability led to impaired spatial learning, which was abolished when TREK-2 was knocked down. Knockdown did not alter the resting membrane potential of stellate neurons in the entorhinal cortex.

In another study, norepinephrine was shown to activate TREK-2 and thereby hyperpolarize neurons at rest in the superficial layers of the entorhinal cortex. This mechanism appeared to involve *α*-_2A_ adrenergic receptors as well as the G_*α*i_ and PKA pathways [66].

In humans, FHEIG (facial dysmorphism, hypertrichosis, epilepsy, intellectual disability/developmental delay, and gingival overgrowth) was associated with a missense mutation in TRAAK. The recombinant mutant TRAAK channel showed a significant gain of function basally and damaged sensitivity to mechanical stimulus and arachidonic acid [67].

#### 2.2.3. TASKs

TASKs are widely expressed across the central nervous system [10, 68]. TASK-1 may be most abundant in the cerebellum, while TASK-3 may be more abundant in the hippocampus, cortex, cerebellum, and certain nuclei, such as the paraventricular nuclei of the thalamus, locus coeruleus, and the dorsal raphe [11]. TASK electrophysiology has been studied in cerebellar granule neurons of rats and serotonergic raphe neurons of mice [69, 70]. Knocking out TASK-1 from mice did not alter resting membrane potential of cerebellar granule neurons or the properties of their action potentials [71]. In contrast, knocking out TASK-3 depolarized cerebellar granule neurons by 10 mV and decreased both the rheobase and action potential amplitude [72]. These results suggest that TASK-3 may contribute more to the resting membrane potential of cerebellar granule neurons than TASK-1.

In mature neurons, transmembrane chloride gradients are mainly regulated by KCC2 and NKCC1, two cation-chloride cotransporters, which, respectively, mediate outward and inward cotransport of Cl^−^ and K^+^ under physiological conditions. Consistent with this idea, chronic knockdown of KCC2 in rat hippocampus increased neuronal excitability by downregulating levels of TASK-3 at the membrane [73]. Altogether, these studies indicate that TASKs stabilize neuronal membrane potential and regulate their activity. As a result, these channels likely play roles in epilepsy, sleep disorder, and depression, as described below.

*(1) Epilepsy*. In the rat entorhinal cortex, application of serotonin depolarized GABAergic interneurons at rest, increased action potential firing frequency, and increased GABA release [74]. As a result, pyramidal cells showed lower excitability. These effects of serotonin were associated with inhibition of TASK-3 in interneurons, suggesting that the channel may be a target in treating epilepsy. Future research is required to determine whether selective inhibition of TASK-3 can alleviate one or more subtypes of epilepsy.

The *Kcnk9* gene coding for TASK-3 is located at chromosomal position 8q24, a locus associated with absence epilepsy [75, 76], although the association is somewhat controversial [77]. A mutation in TASK-3 has been linked to absence epilepsy in a rat model, but mutant and wild-type animals showed similar leaky K^+^ currents [78]. Further work is needed to clarify whether and how this mutation contributes to absence seizures.

*(2) Sleep Disorder*. Sleep is essential to emotional health, but the molecular processes that determine daily sleep duration and the sleep-wake cycle remain elusive. Thalamocortical neuronal networks alternate between burst activity during sleep and tonic single-spike activity during wakefulness [79]. Inhibition of TASK-1 and TASK-3 depolarized thalamic relay neurons, leading them to engage preferentially in tonic single-spike activity [80, 81]. Similarly, inhibiting TASK-3 using spermine during repetitive activity or hypoxia depolarized the thalamic neurons by about 8 mV and switched the firing mode from burst to tonic [82]. Thus, TASKs have potential roles in the sleep-wake cycle.

Consistent with this idea, animals expressing TASK-3 mutants showed increased nocturnal activity and shorter REM sleep [83, 84]. Animals deficient in TASK-3 lacked an *θ* oscillation that was seen in the cortical electroencephalogram of control animals and that resembled type II *θ*. The deficient animals also progressed much more slowly from wakefulness to sleep, and their sleep episodes and REM *θ* oscillations were more fragmented [79]. Knocking out TASK-3 severely shortened sleep [85]. Exposing mice to chronic sleep fragmentation depolarized medial habenula cholinergic output neurons at rest and increased the frequency of their spontaneous firing. Inhibition of TASK-3 mimicked the sleep fragmentation effects in control mice, while the effects of TASK-3 inhibition were absent in sleep fragmentation mice [86]. These results suggest that TASK-3 mediates the ability of sleep fragmentation to increase the excitability of medial habenula cholinergic output neurons.

These findings identify TASK-3 as a mediator of sleep disturbance, but how TASK-3 regulates firing patterns is still unclear. Further study is needed to investigate how TASK-3 contributes to the normal sleep cycle and how its impairment contributes to emotional disorders related to sleep disturbance, such as depression and anxiety.

*(3) Depression*. Knocking out TASK-3 from mice impaired working memory, based on the T-maze spontaneous alternation test, and it damaged spatial memory, based on the Morris water maze test [83]. At the same time, TASK-3 knockout alleviated depressive behaviors, as assessed in the forced swimming test and tail suspension test [84]. These observations identify TASK-3 as a potential target in the treatment of depression. In fact, the receptor may help mediate the antidepressive effects of cannabinoid receptor agonists in mouse models [87–89]: knocking out TASK-3 rendered the animals less responsive to the agonist WIN55212-2 mesylate [83]. Future research is needed to validate how TASK-3 regulates depression.

Dysfunction of TASK-3 was also identified to cause neurological disorders in humans. A missense mutation in TASK-3 was identified to abolish the channel's currents and resulted in a maternally transmitted genomic-imprinting syndrome characterized by mental retardation, hypotonia, and dimorphisms in patients [90, 91].

#### 2.2.4. Tandem of Pore Domains in a Weak Inward Rectifying K^+^ Channel (TWIK)

TWIK-1 appears to be expressed widely across the central nervous system [92], but few studies have examined its physiological roles in neurons. In the entorhinal cortex, serotonin activated TWIK-1, which hyperpolarized stellate and pyramidal neurons at rest in superficial layers, and it slowed their action potential firing [93]. It is unclear whether TWIK-1 in the entorhinal cortex acts as a homodimer or as a heterodimer with another K2P channel isotype.

In dentate gyrus granule cells, downregulating TWIK-1 depolarized the cells at rest and increased their firing rate [94]. TASK-3 appeared to localize in the proximal dendrites and soma of those cells, similar to the localization of TWIK-1. Knocking down both TWIK-1 and TASK-3 depolarized the cells at rest and increased their action potential firing rate [95]. These results suggest that TWIK-1 and TASK-3 contribute to the intrinsic excitability of dentate gyrus granule cells.

## 3. K2P and the Potassium Homeostasis of Glia

Glia support neuronal functions *via* various mechanisms in the central nervous system, and K2P channels are expressed in glia. A transcriptome analysis detected the mRNAs encoding TWIK-1 and TREK-1 in astrocytes [96], and immunohistochemistry detected TASK-1 protein in astrocytes of hippocampus and oligodendrocytes in the mouse brain [97]. Several studies have examined the functions of K2P channels in glia (Figure 2), though much more work remains to be done.

### 3.1. Astrocytes

Astrocytes are star-shaped, specialized glia that differentiate from a neural stem cell pool. They primarily mediate ionic homeostasis and provide structural support to neurons. They also contribute to neurotransmitter release and synaptic development. Astrocyte dysfunction, termed reactive astrogliosis, is common in injury or disease affecting the central nervous system, including some neurological diseases related to aging: Huntington's disease, Alzheimer's disease (AD), and Parkinson's disease [98].

#### 3.1.1. Astrocyte and Passive Conductance

TREK-1 and TWIK-1 in astrocytes contribute to the large K^+^ conductance of cultured cortical astrocytes [13]. While knocking out TWIK-1 from mice did not significantly alter astrocytic passive conductance [99], depleting it from cultured astrocytes led to more negative resting membrane potential [100]. In contrast, eliminating TREK-1 from cultured astrocytes did not alter either resting membrane potential or passive potassium conductance [99]. On the other hand, treating cultured astrocytes or hippocampal slices with spadin reduced their passive conductance by inhibiting the activity of TWIK-1/TREK-1 heterodimers [101]. These data suggest that TREK-1 mildly contributes to the passive conductance of astrocytes. Knocking out TREK-1 alone or together with TWIK-1 did not alter the expression of other potassium channels, suggesting that the other channels do not compensate for K2P channel activity [102].

#### 3.1.2. Glutamate Release

Glutamate is one of the most important neurotransmitters that are released from excitatory presynaptic neurons and that mediate neuronal transmission. Emerging evidence suggests that astrocytes can also release glutamate to modulate synaptic plasticity, neuronal excitability, and transmitter release in physiological and pathophysiological states [103–105]. How astrocytes release glutamate is controversial: for example, does it depend on channels, transporters, or vesicular exocytosis? Several studies in cultured astrocytes and brain slices suggest that increased intracellular Ca^2+^ concentration induces glutamate release *via* a pathway involving the exocytotic machinery [106]. However, these findings may not be entirely reliable, given that the supporting experiments relied on nonspecific methods to manipulate intracellular Ca^2+^ concentration and glutamate transport in astrocytes. For example, the rise of intracellular Ca^2+^ also activates the Ca^2+^-activated K^+^ channels in astrocytes and extrudes K^+^ onto surrounding neurons, depolarizing them and probably resulting in glutamate release [106]. Using a metabotropic glutamate receptor agonist to induce a change in Ca^2+^ concentration in astrocytes may also trigger other changes due to activation of the same receptor on neurons. Indeed, pharmacological inhibition of certain anion channels can reduce glutamate release from astrocytes independently of the exocytotic machinery [103].

Brief synaptic activity can trigger K^+^ uptake via K2P channels containing TREK-1, leading to a temporary increase in astrocyte volume. The volume returns to normal when bestrophin-1 allows Cl^−^ out of the cell [107]. TREK-1 is responsible for fast glutamate release from astrocytes, and the Ca^2+^-activated Cl^−^ channel bestrophin-1 is responsible for slow glutamate release [108]. Fast release depends on activation of G_*α*i_ and dissociation of G_*βγ*_, followed by interaction between G*_*βγ*_* and TREK-1, leading to the opening of glutamate-permeable TREK-1 [109]. This overall pathway is mediated by G_i_-type G protein-coupled receptors including cannabinoid receptor 1, adenosine receptor A1, and *μ*-opioid receptor. Like TREK-1, TWIK-1 can directly interact with the G_*γ*_ subunit; in the presence of G_*γ*_, the heterodimer TWIK-1/TREK-1, which is expressed in astrocytes [13, 96, 100, 110], becomes permeable to glutamate and K^+^ [100]. These data suggest that G_*βγ*_ alters the pore region of the K2P channel. Further studies are needed to determine how TWIK-1/TREK-1 heterodimers are permeable to glutamate.

#### 3.1.3. Ischemia

Astrocytic TREK-1 is also involved in the pathology of ischemia. In an animal model of focal ischemia induced by arterial occlusion, reperfusion was associated with upregulation of TREK-1 [111]. Inhibition of TREK-1 impaired astrocytic clearance and exacerbated inflammation after ischemia, resulting in neuronal apoptosis [112]. During ischemia, the ischemic metabolite lactate upregulates TREK-1 in astrocytes via the PKA pathway [113]. Astrocyte elevated gene-1 (AEG-1) also induces TREK-1 expression in astrocytes during ischemia [114].

These observations suggest that K2P channels, particularly TREK-1, may be involved in passive K^+^ conductance, glutamate release, and ischemia pathology. Future work, such as using conditional knockout mice, should focus on establishing which K2P channels are expressed in astrocytes and what are their functions.

### 3.2. Microglia

Microglia fulfill various functions within the central nervous system, which are related mainly to immune responses and homeostasis. Microglia monitor for damage, injury, or disease, and they contribute to synaptic pruning. Disruption of microglial function leads to several diseases, and microglia-mediated neuroinflammation, associated with synaptic loss and cognitive decline [115], is a characteristic of late AD. Dysregulation of synaptic pruning is associated with cognitive defects in autism [116].

Little is known regarding the expression and function of K2P channels in microglia. THIK-1 appears to play a critical role in microglial ramification and immune surveillance in the brain [117]. Microglia in rat brain slices showed a more depolarized potential (-40.6 mV) and higher input resistance than neurons or other glia. Locally puffing 100 mM ATP onto microglia activated the P2Y_12_ receptor and hyperpolarized their membrane by ~30 mV [117]. Pharmacological blockade or genetic ablation of THIK-1 depolarized the microglia at rest, inhibiting microglial ramification and surveillance. These results suggest that by modulating resting membrane potential of microglia, THIK-1 may help regulate microglia function. Blocking THIK-1 in brain slices with quinine, bupivacaine, or tetrapentylammonium abolished the release of interleukin-1*β* from microglia [117]. This implies that THIK-1 may be involved in the pathological function of microglia in brain diseases. Future studies should investigate the link between resting membrane potential and microglial function, especially since the resting membrane potential of microglia varies across brain regions. Future research should also clarify the region and cell type specificity of THIK-1 expression in the brain.

### 3.3. Oligodendrocytes

The primary function of oligodendrocytes is to insulate the neuronal axon by creating a myelin sheath, which contributes to rapid signal transduction. TASK-1 can induce currents in oligodendrocytes in response to extracellular acidification, and lack of TASK-1 depolarizes oligodendroglial precursor cells [97]. Inhibition of TASK-1 can protect oligodendrocytes from ischemic injury [118]. TASK-1 may be involved in multiple sclerosis, but the details are unclear. In multiple sclerosis, insufficient recruitment and differentiation of oligodendroglial precursor cells leads to incomplete remyelination. The demyelination and inflammation in the central nervous system of the mice model of multiple sclerosis was alleviated by treatment with bupivacaine, which acts against TASK-1 as well as other channels [97], but not by specific TASK-1 knockout or inhibition [119, 120]. In another mouse model of multiple sclerosis, TASK-1 knockout increased the number of mature oligodendrocytes and accelerated developmental myelination, yet it did not affect oligodendroglial differentiation during remyelination after pathological demyelination [97]. Future studies should clarify whether and how TASK-containing K2P channels help mediate oligodendroglial differentiation and remyelination in normal and disease contexts.

## 4. Future Perspectives

K2P channels, which differ from K_v_ and K_ir_ channels, produce leaky K^+^ currents to regulate neuronal excitability. K2P channels are distributed widely in the peripheral and central nervous systems, where their expression patterns vary. In neurons, K2P channels modulate resting membrane potential and action potentials, allowing them to influence multiple biological functions. In the peripheral nervous system, TRESK, TASK, and TREK are involved in nociception and pathological pain. Activation of these channels reduces neuronal excitability and thereby alleviates allodynia in several animal models of pain, suggesting that these channels might be potent targets in pain management. In the central nervous system, activation of G protein-coupled receptors induces K2P channels to regulate neuronal excitability in ways that can influence chronic pain, ischemia, epilepsy, sleep disorder, and major depressive disorder.

While neuronal K2P channels regulate excitability, glial K2P channels are likely more involved in K^+^ and neurotransmitter homeostasis. TREK-1, TWIK-1, THIK-1, and TASK-1 are thought to be expressed in glia, but further investigation is needed to confirm the regional and cell-type distribution of K2P channels. Indeed, much more work is needed to elucidate the functions of K2P channels in glia as well as in neurons, such as through animal studies in which channels are knocked out of specific cell types under physiological and pathological conditions.
